# Application of a high-resolution melt assay for monitoring SARS-CoV-2 variants in Burkina Faso and Kenya

**DOI:** 10.1128/msphere.00027-25

**Published:** 2025-05-29

**Authors:** Caitlin Greenland-Bews, Sonal Shah, Morine Achieng, Emilie S. Badoum, Yaya Bah, Hellen C. Barsosio, Helena Brazal-Monzó, Jennifer Canizales, Anna Drabko, Alice J. Fraser, Luke Hannan, Sheikh Jarju, Jean-Moise Kaboré, Mariama A. Kujabi, Cristina Leggio, Maia Lesosky, Jarra Manneh, Tegwen Marlais, Julian Matthewman, Issa Nebié, Eric Onyango, Alphonse Ouedraogo, Kephas Otieno, Samuel S. Serme, Sodiomon Sirima, Ben Soulama, Brian Tangara, Alfred Tiono, William Wu, Emily R. Adams, Abdul Karim Sesay, Chris Drakeley, Feiko O. ter Kuile, Issiaka Soulama, Simon Kariuki, David J. Allen, Thomas Edwards

**Affiliations:** 1 The Department of Tropical Disease Biology, Liverpool School of Tropical Medicine 9655, Liverpool, United Kingdom; 2 Department of Infection Biology, Faculty of Infectious and Tropical Diseases, London School of Hygiene and Tropical Medicine 270390, London, United Kingdom; 3 Centre for Global Health Research, Kenya Medical Research Institute 118982, Kisumu, Kisumu County, Kenya; 4 Groupe de Recherche Action en Santé (GRAS) 576493 , Ouagadougou, Burkina Faso; 5 Medical Research Council Unit The Gambia at the London School of Hygiene and Tropical Medicine 47969 , Fajara, Gambia; 6 National Heart and Lung Institute, Imperial College London 90897, London, United Kingdom; 7 Quantitative Engineering Design (QED), Sheridan, Wyoming, USA; 8 Department of Clinical Sciences, Liverpool School of Tropical Medicine 574708, Liverpool, United Kingdom; 9 UK-Public Health Rapid Support Team (UK-PHRST), UK Health Security Agency (UKHSA) 371011, London, United Kingdom; 10 Faculty of Epidemiology and Population Health, London School of Hygiene and Tropical Medicine 4906, London, United Kingdom; 11 Global Access Diagnostics (GADx) 723729 , Bedford, United Kingdom; 12 Institut de Recherche en Sciences de la Santé (IRSS) 307955, Ouagadougou, Burkina Faso; 13 Department of Comparative Biomedical Sciences, Section Infection and Immunity, Faculty of Health and Medical Science, University of Surrey 105648 , Guildford, United Kingdom; University of Saskatchewan, Saskatoon, Saskatchewan, Canada

**Keywords:** SARS-CoV-2, variants of concern, HRM, diagnostics, surveillance, Burkina Faso, Kenya, Africa, COVID-19

## Abstract

The rapid emergence and global dissemination of severe acute respiratory syndrome coronavirus 2 (SARS-CoV-2) highlighted a need for robust, adaptable surveillance systems. However, financial and infrastructure requirements for whole-genome sequencing mean most surveillance data have come from higher-resource geographies, despite unprecedented investment in sequencing in low- and middle-income countries (LMICs). Consequently, the molecular epidemiology of SARS-CoV-2 in some LMICs is limited, and there is a need for more cost-accessible technologies to help close data gaps for surveillance of SARS-CoV-2 variants. To address this, we have developed two high-resolution melt (HRM) curve assays that target variant-defining mutations in the SARS-CoV-2 genome, which give unique signature profiles that define different SARS-CoV-2 variants of concern (VOCs). Extracted RNA from SARS-CoV-2-positive samples collected from 205 participants (112 in Burkina Faso, 93 in Kenya) enrolled in the MALCOV study (Malaria as a Risk Factor for COVID-19) between February 2021 and February 2022 were analyzed using our optimized HRM assays. With next-generation sequencing on Oxford Nanopore MinION as a reference, two HRM assays, HRM-VOC-1 and HRM-VOC-2, demonstrated sensitivity/specificity of 100%/99.29% and 92.86%/99.39%, respectively, for detecting Alpha, 90.08%/100% and 92.31%/100% for Delta, and 93.75%/100% and 100%/99.38% for Omicron BA.1. The assays described here provide a lower-cost approach to conducting molecular epidemiology, capable of high-throughput testing. We successfully scaled up the HRM-VOC-2 assay to screen a total of 506 samples from which we were able to show the replacement of Alpha with the introduction of Delta and the replacement of Delta by the Omicron variant in this community in Kisumu, Kenya.

**IMPORTANCE:**

The rapid evolution of the severe acute respiratory syndrome coronavirus 2 variants of concern (VOCs) demonstrated the need for accessible surveillance tools so all communities can conduct viral surveillance. Sequencing, the gold standard, is still a largely inaccessible methodology in low-resource settings. Here, we present a quick, low-cost tool to screen for the common VOCs, designed to support surveillance efforts in low-resource settings. This tool was used to screen samples from Burkina Faso and Western Kenya throughout the pandemic. We show through comparison to sequencing that our assay can generate highly similar data on the different variants circulating in a population, therefore showing the effectiveness of our tool. While not a replacement for sequencing, we present a method of screening and prioritizing samples for further investigation and reduce overburdening sequencing capacity. Our findings provide insight into one potential tool that could be further applied to pathogen screening in the absence of robust sequencing infrastructure.

## INTRODUCTION

As the coronavirus disease 2019 (COVID-19) pandemic progressed, the evolution of severe acute respiratory syndrome coronavirus 2 (SARS-CoV-2) gave rise to variants of concern (VOCs). These VOCs posed an increased and significant threat to the global population and jeopardized public health measures and interventions that had been deployed (1). Detection and surveillance of these variants were primarily achieved through sequencing, which was crucial for tracking the spread of the VOCs worldwide. Genomic surveillance is only beneficial when it is representative spatially and temporally (2), and while many countries benefitted from real-time genomic surveillance during the COVID-19 pandemic, most genomic information of SARS-CoV-2 is from higher-income countries (3).

As of September 2021, 18 months into the COVID-19 pandemic, sequences originating from Africa accounted for approximately 1% of the total 3.5 million sequences available (4). Similarly, it was found that as of October 2021, high-income countries were uploading 12 times more sequences than low- and middle-income countries (2). As of March 2022, there were 100,000 SARS-CoV-2 sequences available from African countries. This represented an incredible milestone in genomic surveillance in Africa and is the result of huge investments to increase sequencing capacity, with SARS-CoV-2 sequences far outnumbering any number of pathogen sequences submitted before from the continent (2). Although investments in sequencing infrastructure are ongoing, this surveillance gap highlights the need for more accessible surveillance methods to be developed and utilized in the interim. Molecular diagnostics offer a viable alternative for targeting SARS-CoV-2 VOCs that are highly sensitive.

One promising method is high-resolution melt (HRM) assays, which feature a post-PCR analysis method that is highly sensitive in detecting nucleotide changes from shifts in amplicon melting temperature. This method has been used to identify individual mutations (5–9) with high sensitivity for detecting their respective targets. The broad range of mutations targeted across the literature includes the VOC-specific mutations N501Y, D614G, L452R, and K417N/T (7–9). However, many of these assays must be run simultaneously in singleplex to allow differentiation between multiple VOCs. This increases the work time, cost of reagents, and the volume of valuable samples required for genotyping. A one-step HRM that could identify multiple mutations in one assay while cutting down on cost and time would be ideal.

Here, we build upon our previous work of one such HRM assay capable of identifying Alpha, Beta, Gamma, Delta, and Omicron VOCs (10). We have developed our toolkit approach further, expanding the available primer sets and developing a new assay that targets the Alpha, Delta, and Omicron (BA.1) VOCs. We evaluate both assays’ ability to detect Alpha, Delta, and Omicron (BA.1) VOCs and compare our HRM results against next-generation sequencing (NGS) from Oxford Nanopore MinION (MK1B, Oxford, UK). This evaluation was conducted using samples collected in Burkina Faso and Kenya from February 2021 to February 2022.

## MATERIALS AND METHODS

### Sample collection and study setting

All samples were collected as part of the Malaria as a Risk Factor for COVID-19 in Western Kenya and Burkina Faso (MALCOV) study (NCT04695197).  Mid-nasal swabs were taken from SARS-CoV-2-positive participants and stored in viral transport media (Biocomma). Samples were collected between February 2021 and February 2022. Details of the study settings and sites involved can be found in the study protocol (11). This study was conducted across locations in the United Kingdom and sub-Saharan Africa. Assays were developed and validated in the United Kingdom, training was conducted in Kenya and Gambia, and testing was conducted in Kenya, Gambia, Burkina Faso, and the United Kingdom. One hundred twelve samples from Burkina Faso and 93 from Kenya were sequenced and analyzed by both HRM assays (HRM-VOC-1 and HRM-VOC-2). A further 413 samples from the Kenyan cohort were analyzed by HRM-VOC-2 (total sample count analyzed by HRM-VOC-2, *n* = 506) but were not sequenced to determine the molecular epidemiology of the variants of concern.

### RNA extraction

RNA was extracted from clinical specimens in viral transport media (VTM) using the QIAamp Viral RNA Kit (QIAGEN, Germany), following the manufacturer’s protocol, and implemented as an automated workflow using the QIAcube HT platform (QIAGEN, Germany). Purified RNA was eluted in 50 µL of elution buffer and stored at −80°C until use.  

### Reverse transcription-polymerase chain reaction (RT-PCR)

RT-PCR was performed by staff on-site in Kenya and Burkina Faso according to the study protocol.

### Design of HRM-VOC-2 assay

Sequences representing the known variants classified by the World Health Organization as variants of concern (VOC), under monitoring (VUM), and of interest (VOI) were downloaded from GenBank and aligned using ClustalX in BioEdit (version 7.2.5). Lineage-defining mutations were identified from the literature and online repository https://covariants.org/ (12) and located within the alignment.

Primers were designed (Table S1) with the aid of Primer 3 (13), and where no suitable primers could be obtained, primers were designed manually. The suitability of primers was initially tested *in silico* using OligoCalc (14) and uMelt (15) to ensure compatible melting temperatures (Tms).

Singleplex testing was conducted during assay development to ensure specificity of primers and was conducted by testing each primer pair on extracted RNA from cultured viral isolates for Alpha (GenBank accession number: MW980115), Beta (hCoV-19/South Africa/KRISP-EC-K005321/2020) (BEI Resources), Gamma (hCoV-19/Japan/TY7-503/2021), Delta (SARS-CoV-2/human/GBR/Liv_273/2021), Omicron (BA.1) (SARS-CoV-2/human/GBR/Liv_1326/2021), and wild type (isolate REMRQ0001/Human/2020/Liverpool) (Alpha/Beta/Gamma/Delta/OmicronBA.1/OmicronBA.2/WT), and following this, a multiplex was formed with compatible peak Tms that targeted Alpha, Delta, and Omicron (BA.1). No further testing was possible with Beta and Gamma variants due to their absence in the clinical sample sets.

### HRM assays

Two multiplex HRM assays were evaluated, each containing four different primer pairs, the HRM-VOC-1 assay as described in (10) and the HRM-VOC-2 assay described above. For each assay, 2.5 µL of RNA template was added for 12.5 µL final reaction volumes using Lunar Universal Probe One-Step RT-qPCR kit (New England BioLabs, USA), 1× EvaGreen dye (Biotium, USA), and primers added to their optimized concentrations (Table 1).

**TABLE 1:** Optimized final reaction primer concentrations for primer set in the two multiplex assays (HRM-VOC-1 and HRM-VOC-2)

Assay	Mutation targeted	Associated variants	Final forward primer concentration (nM)	Final reverse primer concentration (nM)
HRM-VOC-1	S_del. 156–157	Delta	100	100
	S_K417N	Beta/Gamma/Omicron BA.1	150	150
	N_D3L	Alpha	600	600
	S_EPE	Omicron BA.1	250	250
HRM-VOC-2	S_A570D	Alpha	400	400
	S_L452R	Delta	200	200
	S_EPE	Omicron BA.1	400	400
	Orf1b_Control	All	100	100

Reactions were performed using QuantStudio 5 (Thermo Fisher, USA) for Kenyan samples and QuantStudio 6/7 flex (Thermo Fisher, USA) for Burkinabe samples. The thermal cycle profiles are found in Table S2.

### Analysis of HRM assay data

Data were visualized as negative first derivative plots using QuantStudio Design and Analysis Software (v.1.5.2, QuantStudio 5 systems, Thermo Fisher Scientific Inc.).  

**Fig 1:**
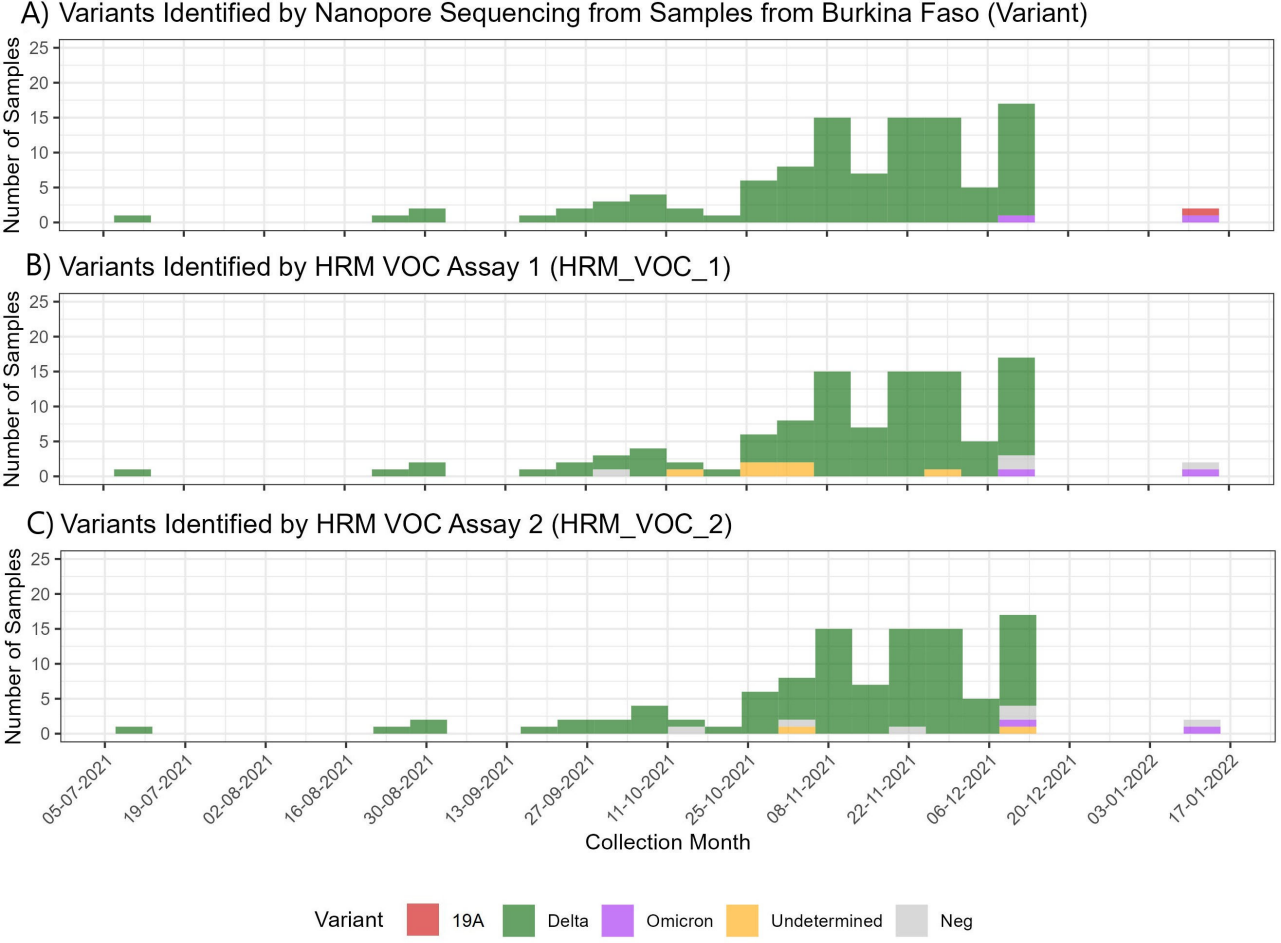
SARS-CoV-2 variants identified in Burkina Faso using different methods. (**A**) Number of samples collected in Burkina Faso from July 2021 to January 2022 and the variants that were identified by nanopore sequencing. (**B**) Number of samples in the Burkina Faso cohort and the variant identified by using the HRM-VOC-1 assay. Negative results represent those with no amplification observed; undetermined samples had amplification but no identifiable VOC peak. (**C**) Number of samples in the Burkina Faso cohort and the variant identified by using the HRM-VOC-2. Negative results are those where no amplification was observed, and undetermined results are those with a control peak without an identifiable VOC peak.

**Fig 2:**
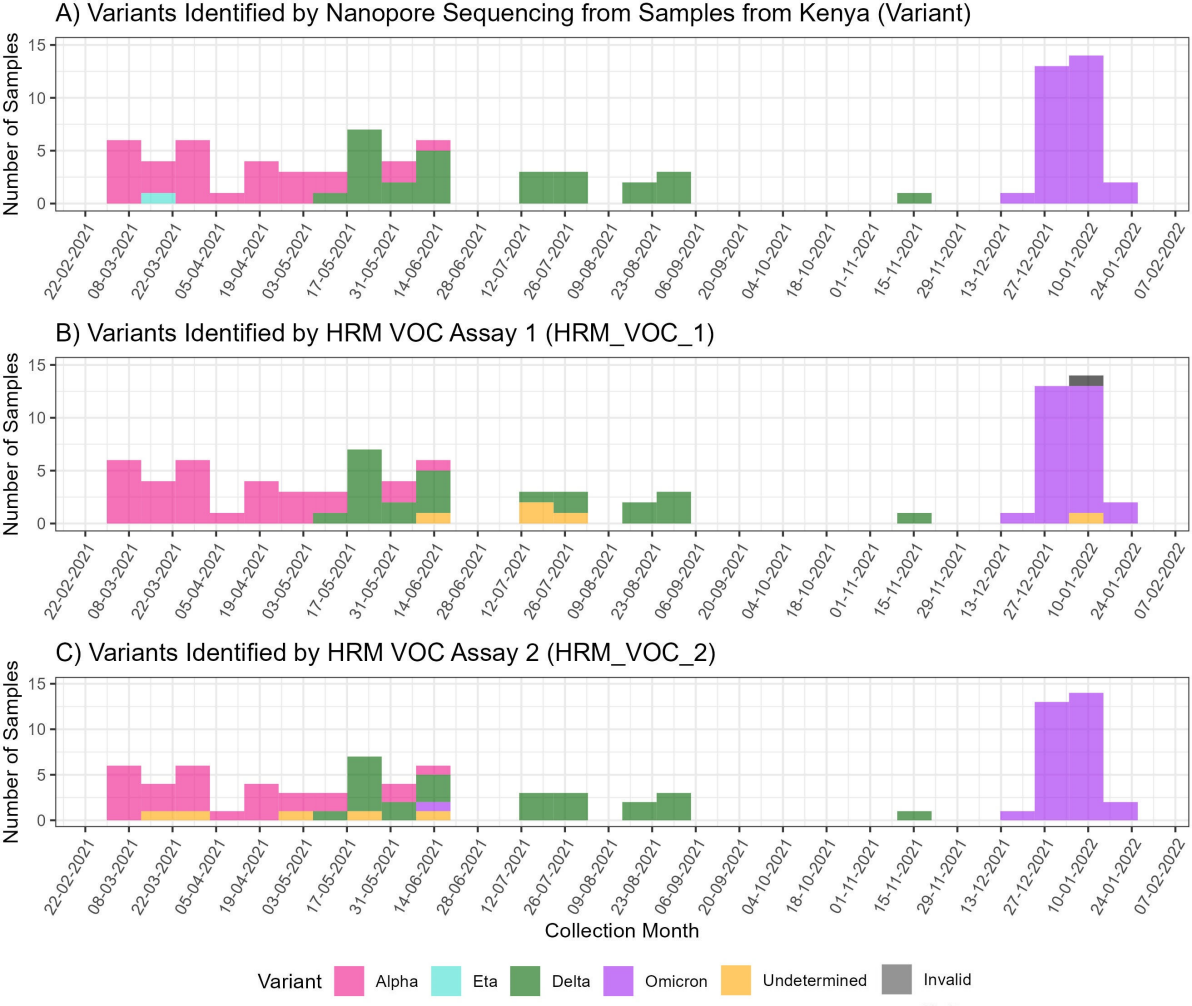
SARS-CoV-2 variants identified in Kenya using different methods. (**A**) Number of samples collected in Kenya from February 2021 to February 2022 and the variants that were identified by nanopore sequencing. (**B**) Number of samples in the Kenyan cohort and the variant identified by using the HRM-VOC-1 assay. Negative results represent those with no amplification observed; undetermined samples had amplification but no identifiable VOC peak. (**C**) Number of samples in the Kenyan cohort and the variant identified using the HRM-VOC-2. Negative results represent those with no amplification observed; undetermined samples had amplification but no identifiable VOC peak.

Samples that did not yield enough sequence coverage by nanopore sequencing to identify a variant using NextClade (16) were excluded from further analysis. Samples that gave an HRM peak that could not be assigned to a variant were classified as undetermined. In the instance of HRM-VOC-2, where there is a control peak, if the control peak was absent, these samples were classified as invalid. For HRM-VOC-2, if there is a control peak but the remaining peaks do not fit the signature peaks for the variants of concern and therefore cannot be assigned, these samples were classified as undetermined. For analysis of the assay performance, invalid HRM results were excluded (Fig. 1 and 2). Sensitivity and specificity analysis was performed in the MedCalc diagnostics calculator (17).

### Sequencing

Two hundred five SARS-CoV-2 samples from Burkina Faso and Kenya combined were prepared according to the Artic SARS-CoV-2 sequencing protocol (18). Amplicon generation was conducted using Artic vV.4.1 primers (Integrated DNA Technologies, USA), using Q5 Hot Start High-Fidelity 2× Master Mix (New England Biolabs, USA), 10 µM primer pools, and a thermocycling profile of 30 seconds of 98°C heat inactivation, followed by 25 cycles of 15 seconds of denaturation at 98°C and 5 minutes of annealing/extension at 65°C. Library preparation was carried out using the Ligation Sequencing Kit (SQK- LSK109) and Native Barcoding Expansion Kits (EXP-NBD196, Oxford Nanopore Technologies, UK). Enzymes for barcode and adapter ligation were acquired from New England Biolabs (USA), and AMPure XP beads were acquired from Fisher Scientific (USA). Sequencing was performed on an R.9.4.1 flow cell on a MinION Mk1B device (Oxford Nanopore Technologies, UK) for Kenyan samples and GridION device for Burkinabe samples. All sequences have been deposited on the Sequence Read Archive under the BioProject numbers PRJNA1095865 and PRJNA1096688 for sequences from Kenya and Burkina Faso, respectively.

### Sequencing analysis/bioinformatics

Bioinformatics analysis was performed by following the Artic bioinformatics pipeline (v.1.1.0) (19). Basecalling was performed using Guppy, and a consensus sequence was generated. Consensus sequences were processed by NextClade (v.2.14.1) (16) for rapid variant calling and mutation summaries.

### **Statistical analysis and data processing ** 

#### Diagnostic accuracy 

Samples that did not yield enough coverage from sequencing for a variant to be identified were excluded from analysis due to the lack of a reference standard.

Sensitivity, specificity, and accuracy were calculated for each variant by comparison to the reference standard NGS, and the calculation was performed using MedCalc (17). True positives were defined as samples where the HRM-identified variant matched the variant identified by sequencing. A true negative was every sample correctly identified as a variant other than the target VOC for that analysis. Overall agreement with the NGS result was calculated per assay as the total number of true positives divided by the total number of samples sequenced. Cohen’s kappa (agreement) was calculated and interpreted per variant for each assay as described in McHugh et al. (20).

### Comparison of HRM-VOC-1 and HRM-VOC-2

McNemar’s test was applied to compare the results of HRM-VOC-1 and HRM-VOC-2. This test was performed for each variant (Alpha, Delta, Omicron) using the mcnemar.test() function in R.

### **Data processing and visualization ** 

All data visualization was conducted using R in RStudio (version: 2023.3.1.446). Graphical analysis was undertaken using the ggplot2 package.

## RESULTS

### Variants identified in Kisumu, Kenya

Samples were collected in Kisumu, Kenya, from February 2021 to February 2022 (Fig. 2). In the sample set of 93 samples, nanopore sequencing identified six different clades, 20I (*n* = 28), 21D (*n* = 1), 21A (*n* = 20), 21J (*n* = 5), 21I (*n* = 2), 21K (*n* = 30), corresponding to four different variants, Alpha, Eta, Delta, and Omicron, and six Pango lineages (Fig. S2). Seven samples did not yield high enough quality reads for a variant to be identified using NextClade.

### Detection by HRM

Of the 193 samples with a valid sequencing result from both settings combined, the HRM-VOC-1 assay identified variants in 176 of these (91.2%). One hundred eighteen were identified as Delta, 30 as Omicron, and 28 as Alpha. Eleven samples produced a peak profile, but a variant could not be determined, and five samples showed no amplification and were classified as negative (Fig. 2). The HRM-VOC-2 assay identified variants in 179 of 193 samples (92.7%), 120 were Delta variants, 33 were Omicron, and 26 were Alpha. There was one sample identified as Eta by sequencing; this was identified as a false-positive Alpha result by HRM-VOC-1 and gave a peak classified as “unidentified” by HRM-VOC-2. Seven samples gave a positive HRM result but did not have a peak profile indicative of one of the three targeted VOCs (Alpha/Delta/Omicron), six showed no amplification and were classed as negative, and there was one invalid sample result (Fig. 2). Invalid results were not included in graph visualization or sensitivity or specificity analysis.

### **Assay performance ** 

Sensitivity and specificity were calculated for the combined HRM results across both study locations in comparison with NGS reference (Table 2). One hundred ninety-three samples gave a valid result when using the HRM-VOC-1 assay, and 192 samples when using the HRM-VOC-2 assay. The HRM-VOC-1 assay had a sensitivity and specificity of 100% and 99.39%, respectively, for Alpha, 90.08% and 100% for Delta, and 93.75% for Omicron. The HRM-VOC-2 assay had a sensitivity and specificity of 92.86% and 99.39%, respectively, for Alpha, 92.31% and 100% for Delta, and 100% and 99.38% for Omicron.

**TABLE 2:** Combined performance from Burkina Faso and Kenya of each HRM assay, HRM-VOC-1 and HRM-VOC-2, compared to NGS results

Assay and VOC	True positive	True negative	False positive	False negative	Sensitivity (%) [CI: 95%]	Specificity (%) [CI: 95%]	Accuracy (%)
HRM-VOC-1							
Alpha	28	164	1	0	100 [87.66–100]	99.39 [96.67–99.98]	99.48
Delta	118	62	0	13	90.08 [83.63–94.61]	100 [94.22–100]	93.26
Omicron	30	161	0	2	93.75 [79.19–99.23]	100 [97.73–100]	98.96
HRM-VOC-2							
Alpha	26	164	0	2	92.86 [76.50–99.12]	99.39 [97.78–100]	98.96
Delta	120	62	0	10	92.31 [86.31–96.25]	100 [94.22–100]	94.79
Omicron	32	159	1	0	100 [89.11–100]	99.38 [96.57–99.98]	99.48

### McNemar’s and Cohen's kappa test results

No significant difference was found between the two HRM assays for detecting the three key variants using McNemar’s test (Table 3).

**TABLE 3:** McNemar’s test result from comparing HRM-VOC-1 and HRM-VOC-2 on the combined sequenced sample set

Variant	McNemar’s chi-squared	df	*P*-value
Alpha	1.33	1	0.25
Delta	0.08	1	0.77
Omicron	0.5	1	0.48

There was substantial agreement with the sequencing results for both HRM-VOC-1 and HRM-VOC-2, detecting Delta and Omicron, as Cohen’s kappa was between 0.61 and 0.80, and there was almost perfect agreement with sequencing for Alpha samples (Table 4)(20).

**TABLE 4:** Cohen's kappa test results for comparison between HRM-VOC-1 (A) and HRM-VOC-2 (B) against sequencing results

Assay and variant	Cohen’s kappa
HRM-VOC-1	
Alpha	0.81
Delta	0.68
Omicron	0.79
HRM-VOC-2	
Alpha	0.81
Delta	0.70
Omicron	0.79

### Cycle threshold (Ct) value vs sequencing and HRM success

All samples analyzed were below RT-qPCR Ct 30, with the majority being successfully called by both assays (Fig. S3). One sample gave an invalid result for HRM-VOC-1 with a Ct of 24. Nine samples gave an undetermined result for HRM-VOC-1 with a Ct range of 23.5–29.5, and 10 were undetermined by HRM-VOC-2 and had a Ct range of 23.5–29.5 in both instances.

### Scaling up sample screening by HRM in Kenya

**Fig 3:**
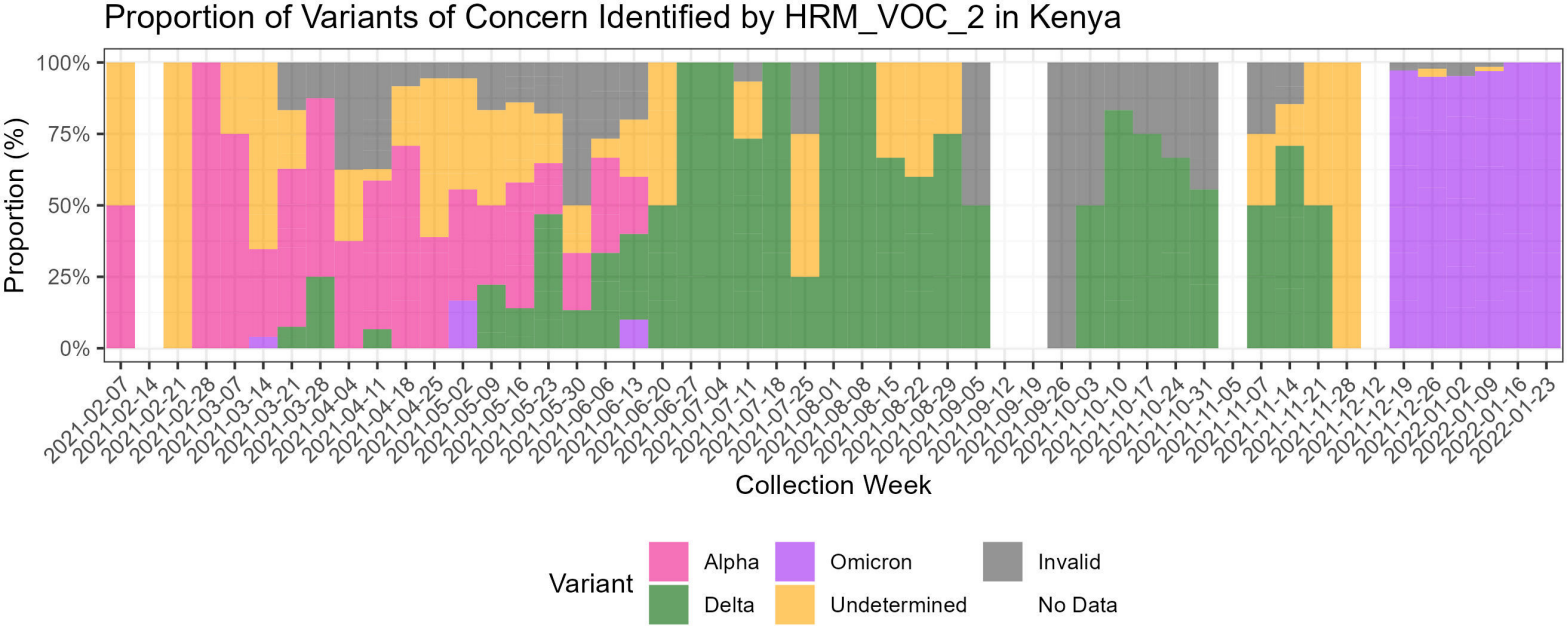
Time series of variants identified by HRM-VOC-2 when tested on 506 SARS-CoV-2 PCR-positive samples collected in Kenya throughout the study period. This is a combined data set including the 86 successfully sequenced samples. Undetermined represents samples that produced a control peak but no identifiable VOC peaks. Invalid represents samples where there was no control peak. No data represents weeks of the year where no positive samples had been collected.

Out of the 506 positive SARS-CoV-2 samples analyzed by HRM-VOC-2, 396 had an identifiable variant (78.3%) (Fig. 3). Of the identifiable variants, 72 samples (18.18%) were identified as Alpha, 98 samples (24.75%) were identified as Delta, and 226 samples (57.07%) were identified as Omicron. Of the remaining 110 samples, 47 gave invalid peak readings (no or limited amplification observed and absence of a control peak), 63 amplified with a control peak, but the other peaks could not be categorized into signature peaks representing the variants of concern and therefore have been labeled as “undetermined” (Fig. 3). Cts were obtained from the MALCOV study team, and it was determined that of these 506 samples, Cts ranged from 17.9 to 39.9, with variants being successfully called across this range (Fig. S4). Samples that could not be called and were labeled as invalid (Fig. 3) all had a Ct of 30 or above, and undetermined samples had a range of 23.5–39.2 (Fig. S4).

Alpha was the dominant variant in the data set at the start of sample collection (7 February 2021) until early May (2 May 2021). From the 2nd of May 2021, the proportion of detected Delta samples increased rapidly. As of 27 June 2021, Delta samples comprised 100% of samples collected. Delta remained the dominant variant detected until the 19th of December when Omicron fully replaced Delta at just below 100% of the total samples analyzed. Three samples were identified as Omicron by HRM-VOC-2 in the first 6 months of the sample set and are represented as Omicron samples in Fig. 3. Due to the timings of these samples being collected, implying it is unlikely that Omicron was circulating at this time, the decision was made to label these three samples as false positives.

## DISCUSSION

Here, we have presented the application of two variant-calling HRM assays to the genotyping of positive SARS-CoV-2 samples in Burkina Faso and Kenya. These assays had high sensitivity in identifying the Alpha, Delta, and Omicron (BA.1) variants of concern when compared to results generated by NGS as the gold standard. Most samples were successfully variant-typed with high concordance to NGS results.

The assay was successfully scaled up to screen more than 500 samples collected over 12 months for the MALCOV study in Kisumu, Kenya. With the HRM assay, we could identify the infecting variant in many of these samples and describe the variant waves in Kisumu during this time. The ability to successfully scale up the HRM demonstrates that if implemented, the HRM can be used for high-throughput analysis of samples that could not be achieved with sequencing. When experiencing high case numbers, this scalability would aid in reducing backlogs and allow prioritization of any existing sequencing infrastructure to be used for samples that were unable to be identified by HRM. Furthermore, the speed of the HRM workflow and analysis is considerably faster than sequencing (a few hours compared to a few days, respectively), which would improve the time to generate crucial data to be used for public health data but would also free the time of laboratory staff for other important tasks.

Our assay has shown three variant replacement events between February 2021 and January 2022, which mirrors the three waves reported during this period from other African countries such as The Gambia (21), Ethiopia (22), and Senegal (23). In the samples analyzed from Kenya in this study, Alpha was the dominant variant between March 2021 and May 2021 and was then replaced by Delta in May 2021, followed by Omicron in mid-December 2021, which is in keeping with epidemiological data from other regions of Kenya (24, 25). Other studies have reported the Beta variant co-circulating with the Alpha variant in regions of Kenya (26). The HRM-VOC-2 assay used to screen all 506 samples in this study does not detect the Beta VOC, and no samples were identified as Beta in those that were sequenced, so it is unknown whether Beta was present in this sample set.

We have demonstrated that HRM is a reliable method of generating epidemiologically important data. HRM assays are also easily scalable, with 506 samples being variant-typed by the HRM-VOC-2 assay. Samples with a Ct lower than or equal to 30 provide the best results for identifying a VOC when analyzing with HRM. When testing the 506 samples, all invalid results for HRM had a Ct greater than 30 (Fig. S4), indicating that samples with lower Ct values should be prioritized where possible to minimize invalid results. Invalid and unidentifiable results when using the HRM are to be expected, even with samples with a Ct lower than 30; however, we recommend that the samples that are either unidentifiable variants or give invalid results with HRM could then be prioritized for NGS. Due to the volume of samples, it would have been expensive and labor-intensive to sequence the total sample set. Based on our results, this would result in only 20% of the total sample set requiring sequencing, reducing the overall expenditure. Finally, the calculated cost of this assay equates to <$1 per sample, compared to the average cost of nanopore sequencing, which has been reported to cost ~$12 per sample when performed at high throughput (19). If scaling testing up to 500 samples using the HRM would cost ~$500 compared to $6,000 for NGS, this would equate to ~$5,500 savings if using HRM.

Several HRM panels for variant identification have been developed throughout the pandemic. An HRM panel for detecting Delta, Omicron BA.1, BA.2, and BA.5 in four separate HRM assays achieved 97.9% agreement with Sanger sequencing (27). Another study in Iran utilized HRM for variant typing due to limited funds available for extensive sequencing and saw 93.68% sensitivity and 100% specificity compared with Sanger sequencing (6). The advantage of the approach presented here is the use of a single-tube assay that can detect Alpha, Delta, and Omicron variants in one reaction without requiring multiple tests, reducing test complexity.

Throughout this study, the assays have been run on multiple instruments when used at different study sites, including QuantStudio 5, QuantStudio 6/7 (Thermo Fisher Scientific Inc, USA), Magnetic Induction Cycler (MIC) (BioMolecular Systems, Australia), and Rotorgene Q (QIAGEN, Germany), highlighting the adaptability of the assays to multiple platforms. The transferability of this assay across platforms negates the need for instrument procurement if deciding to implement this technique, as most modern thermocyclers with the capability to perform HRM can be used.

HRM identified a small number of samples as Omicron in the first 6 months of the sample set. As this is a retrospective sample set, we identified these as probable false-positive results as they pre-date the established date of the first global report of Omicron BA.1 and the date of first detection in Kenya, both occurring in early November 2021 (26, 28). This could be due to non-specific binding of the Omicron primer sets to the RNA, or alternatively, a mutation in one of the primer target sites resulting in a temperature shift of the peak that results in the shift of a peak into the Tm range for Omicron for HRM-VOC-2 resulting in the miscalling of the Omicron variant for these samples. To fully understand these false positives, sequencing would need to be performed to investigate the potential mutations present in the target regions; however, this was outside the scope of this study at the time it was conducted.

The main limitation of this approach, which has also been noted across the literature, is its inability to detect new, emerging mutations, as the assay design relies on pre-existing knowledge of the mutation profile of circulating variants. However, from existing whole-genome sequencing (WGS) surveillance systems, information on novel single nucelotide polymorphisms (SNPs) of novel VOCs can be utilized in the design of HRMs to provide a more agile and accessible assay for more regions to have ownership of their surveillance efforts. In addition to this, unusual peaks from the HRM assays may be observed as a result of new, emerging mutations, and these unusual results can act as a flag for samples to be investigated further by sequencing, allowing the prioritization of samples and avoiding overburdening of existing sequencing infrastructures.

Another limitation lies in the inter-assay variation, which can impact assay interpretation when the assay relies on small shifts in melting temperatures. To improve the analysis of HRM outputs, automation of the process could be used to reduce any user error/unreliability in peak interpretation, which could be achieved through machine learning methods that use previously analyzed data sets to train an algorithm to interpret future outputs (29). This technology could be adapted to provide molecular epidemiological information on other pathogens without the expense of WGS.

While interest in surveillance of SARS-CoV-2 VOCs is waning (as of writing in 2025), this study has demonstrated the power of HRM as a method of conducting surveillance when sequencing infrastructures are limited. Given the relatively straightforward assay design, this method could be adapted and applied to a range of pathogens and be tailored for local epidemiological questions outside of SARS-CoV-2 surveillance. For example, HRM has previously been used for screening for bacteria, antimicrobial resistance genes, and *Plasmodium falciparum* (30–33).

The assays described here are single-tube assays providing results in 3 hours from RNA to variant identification, making them quicker than WGS with far more accessible and streamlined analysis. This technique can make VOC surveillance less costly and more rapid, reducing the wait time from sample to result and reducing reliance and potential overburdening of local and external sequencing infrastructures. This assay’s high sensitivity and specificity have allowed us to investigate the molecular epidemiology of the VOC circulating in Burkina Faso and Kenya during the sample collection windows.

### **Conclusion ** 

HRM provides a quick, low-cost alternative to sequencing that can provide sensitive and specific identification of key mutations in three of the main VOCs of SARS-CoV-2: Alpha, Delta, and Omicron. We have demonstrated that the assays are flexible, easily updatable, and readily applied to retrospective data sets. The use of these assays would not only reduce the cost of genomic surveillance but prevent overwhelming existing sequencing infrastructure during a pandemic or outbreak situation.  

## Data Availability

All sequencing data is deposited in BioProjects : PRJNA1095865 and PRJNA1096688. All other data produced in the present work can be made available upon reasonable request to the authors.

## SUPPLEMENTAL MATERIAL

Supplemental Material msphere.00027-25-s0001.docx Supplemental figures and tables.

ASM does not own the copyrights to Supplemental Material that may be linked to, or accessed through, an article. The authors have granted ASM a non-exclusive, world-wide license to publish the Supplemental Material files. Please contact the corresponding author directly for reuse.
