# Supplementary material for: Application of a high-resolution melt assay for monitoring SARS-CoV-2 variants in Burkina Faso and Kenya
Source: mSphere. 2025 May 29;10(6):e00027-25. doi: 10.1128/msphere.00027-25 (PMC12188703; doi:10.1128/msphere.00027-25)
Supplement: Supplemental Material — Supplemental figures and tables. [file msphere.00027-25-s0001.docx]

**Supplementary Materials**


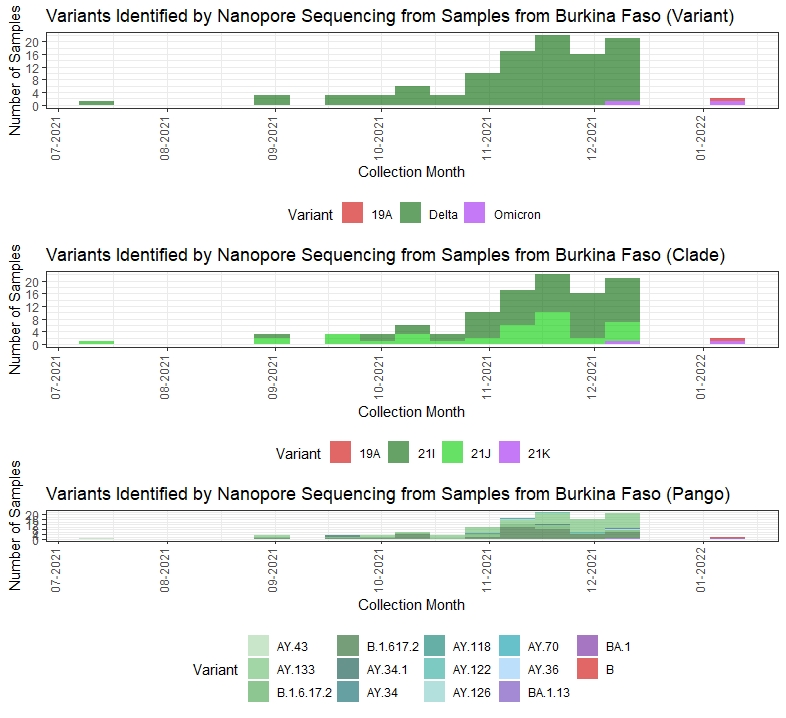


Figure S1: Timeseries of variants (top), Clade (middle) and Pango lineages (bottom) detected by nanopore MinION sequencing in Burkina Faso.


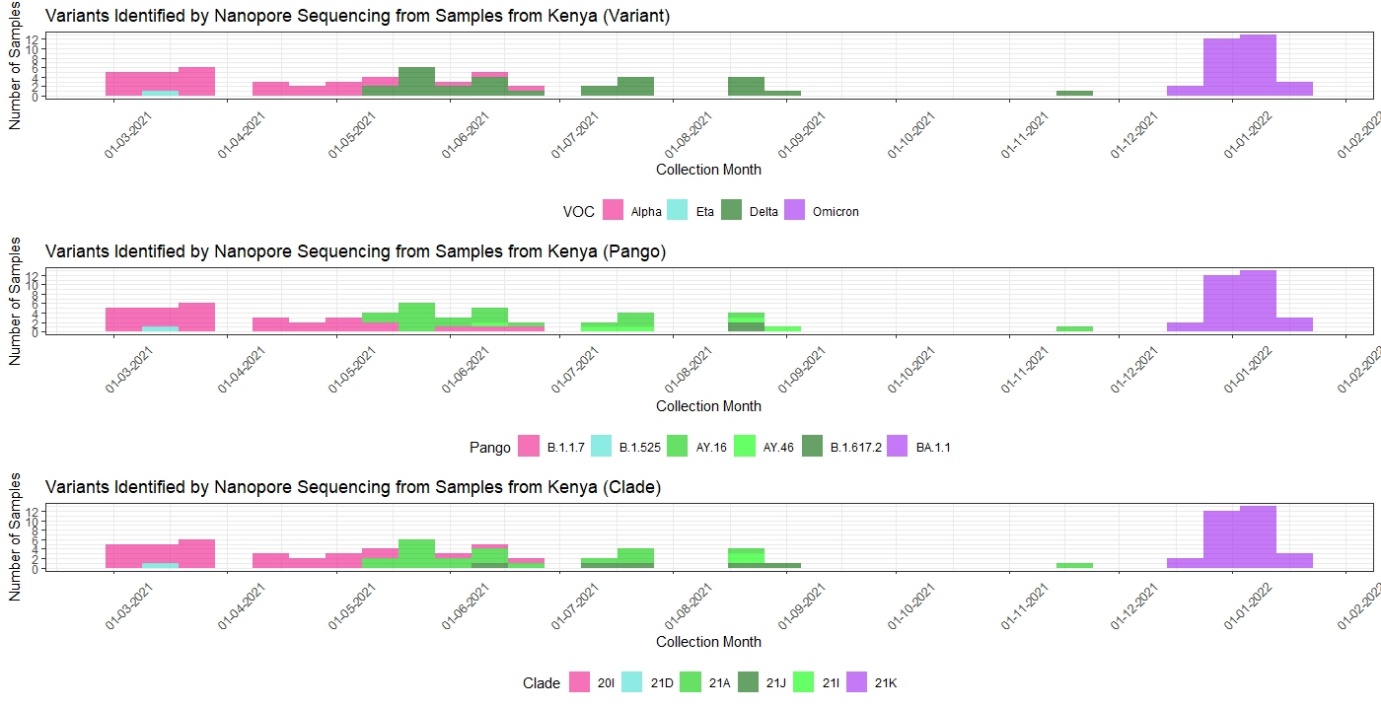


Figure S2:Timeseries of variants (top), Pango lineages (middle) and clades (bottom) detected by nanopore MinION sequencing in Kenya.


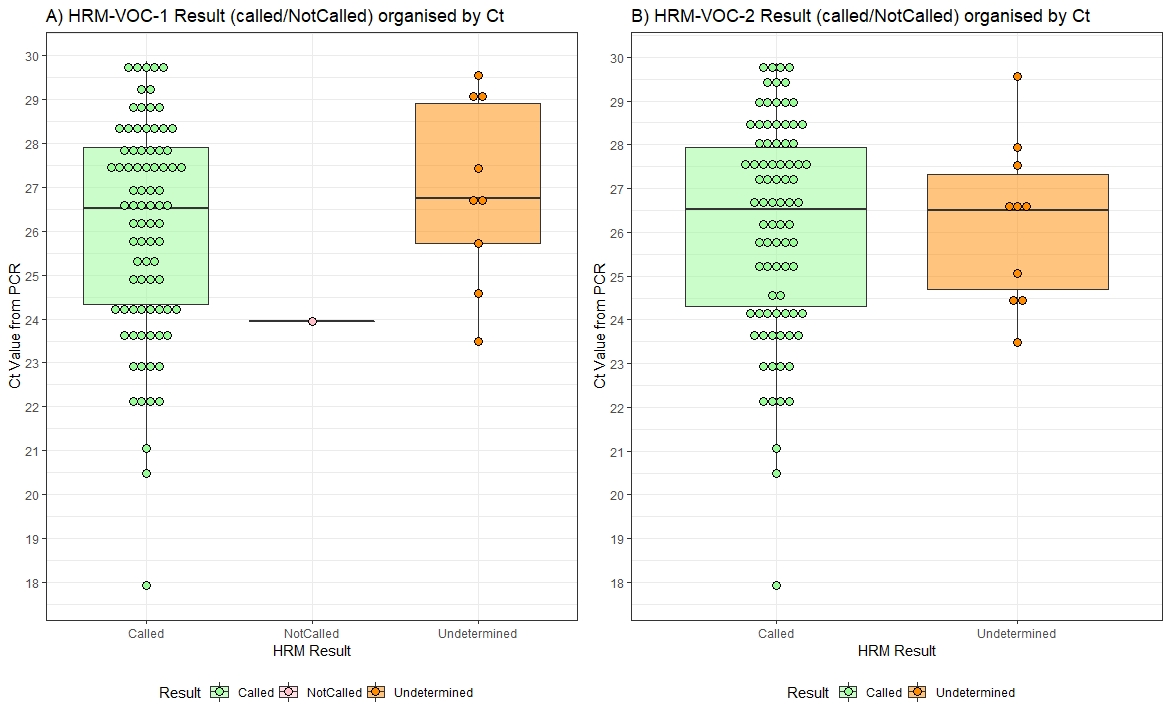


Figure S3: Distribution of samples by Ct from both study sites, separated into whether they could be identified as a VOC (Called), no result or invalid result (NotCalled) and where valid peaks were observed but could not be attributed to a VOC (Undetermined Variant).


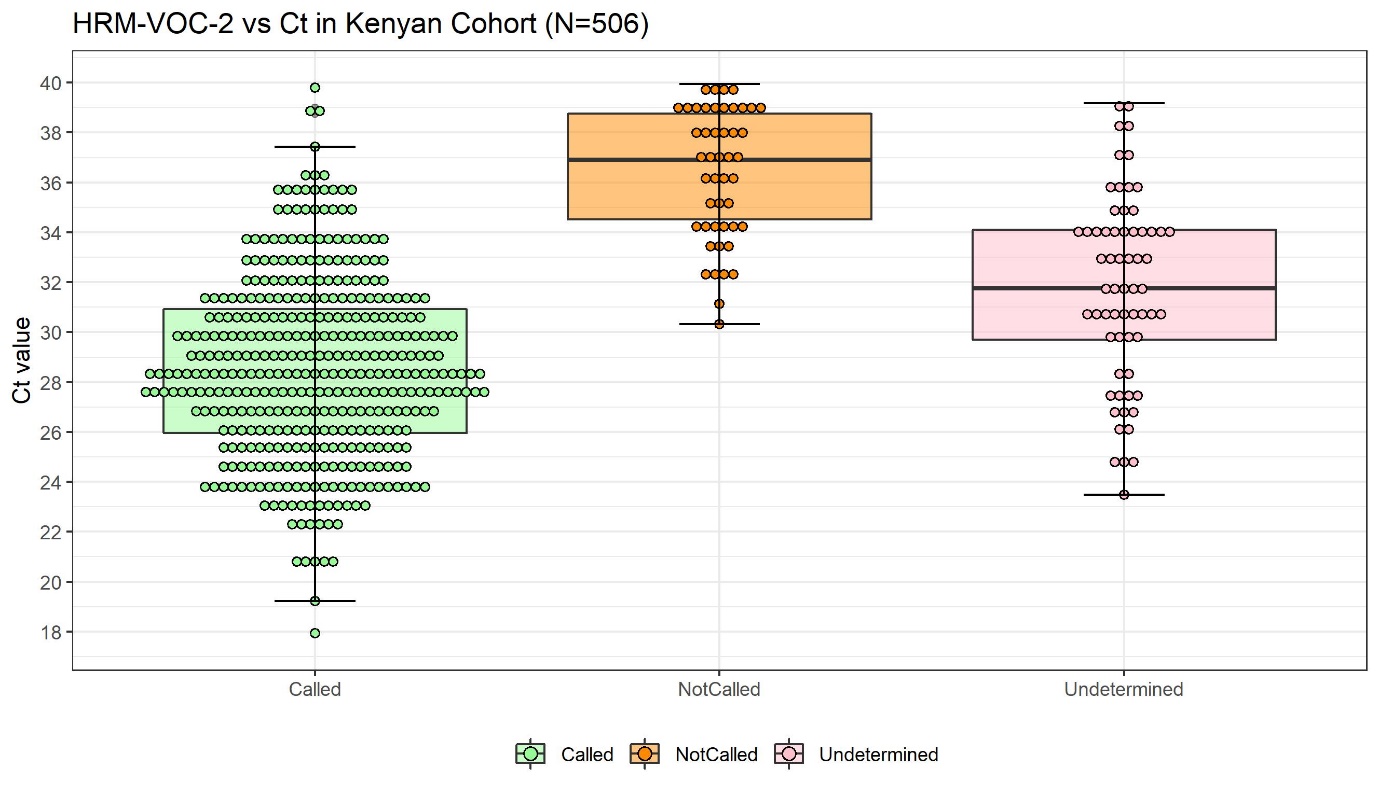


Figure S4: Distribution of samples by Ct from 506 samples from Kenya screened by HRM-VOC-2, separated into whether they could be identified as a VOC (Called), invalid result (NotCalled) and where valid peaks were observed but could not be attributed to a VOC (Undetermined Variant)

| **Table S1;** Description of the SARS-CoV-2 genome targets for, and details of the sequences of the eight primer sets designed to amplify lineage-defining mutations. These primer sets were subsequently incorporated into two multiplex assays: London and Liverpool. Orf1 = Open reading frame 1, S = Spike, N = Nucleocapsid. | | | | | | | | | | | | | | | | | |
| --- | --- | --- | --- | --- | --- | --- | --- | --- | --- | --- | --- | --- | --- | --- | --- | --- | --- |
| **Gene** | **Mutation Targeted** | | **AA- position targeted** | **Primer SetName** | | **Variant** | | | | | | | **Forward Primer Sequence (5’-3’)** | | **Reverse Primer Sequence (5’-3’)** | | |
|  |  |  |  |  |  | **L-Strain** | **Alpha** | **Beta** | **Gamma** | **Delta** | **Omicron** | |  |  |  |  |  |
| S | Substitution | | 570 | S_A570D | |  | X |  |  |  |  | | ATTTGGCAGAGACATTGA | | GCATGAATAGCAACAGGGAC | | |
| S | Substitution | | 452 | S_L452R | |  |  |  |  | X |  | | GATTCTAAGGTTGGTGGTAATTATAATTACCG | | ACCATATGATTGTAAAGGAAAGT | | |
| S | Insertion | | 214 | S_EPE_HRM-VOC-2 | |  |  |  |  |  | X | | ATTATAGTGCGTGAGCCA | | CCAACCTGAAGAAGAATCACCA | | |
| Orf1b | NA | |  | Orf_Control | | X | X | X | X | X | X | | CGTCTGCGGTATGTGGAAAG | | TCCTTTTCTTGGAAGCGACA | | |
| S | Deletion | | 156-157 | S_del. 156-157 | |  |  |  |  | X |  | | ACCACAAAAACA ACAAAAGTTGG | | TTCGCACTAGAA TAAACTCTG AACTC | | |
| S | Substitution | | 417 | S_K417N | |  |  | X | X |  | X | | AAGTCAGACAA ATCGCTCCA | | AACGCAGCCTGT AAAATCATC | | |
| N | Substitution | | 3 | N_D3L | |  | X |  |  |  |  | | AACAAACAA ACTAAAATGTCT G ATA | | TTACTGCCAGTT GAATCTGA | | |
| S | Insertion | | 214 | S_EPE_HRM-VOC-1 | |  |  |  |  |  | X | | CCTATTATAGTGCGTGAGCCA | | CTATGTAAAGCAAGTAAAGTTTGAAACCT | | |
| **Table S2:** HRM Assay thermal cycling conditions | | | | | | | | | | | | | | |  |  |  |
| **Cycling conditions: HRM-VOC-1** | | | | | | | | | | | | | | |  |  |  |
|  | | | **Hold 1 (Reverse Transcription)** | | | | | | | 55°C | | 10min | |  |  |  |  |
|  | | | **Hold 2 (Initial denaturation)** | | | | | | | 95°C | | 1min | |  |  |  |  |
| **Cycling x 40** | | | **Denaturation** | | | | | | | 95°C | | 10 secs | |  |  |  |  |
|  |  |  | **Anneal** | | | | | | | 56°C | | 30 secs | |  |  |  |  |
|  |  |  | **Extend** | | | | | | | 72°C | | 20 secs | | Acquire data |  |  |  |
| **Melt Step** | | | **HRM (0.1°C increment, 2s hold)** | | | | | | | 73°C -85°C | | | | Acquire data |  |  |  |
| **Cycling conditions: HRM-VOC-2** | | | | | | | | | | | | | | |  |  |  |
|  | | | **Hold 1 (Reverse Transcription)** | | | | | | | 55°C | | 10min | |  |  |  |  |
|  | | | **Hold 2 (Initial denaturation)** | | | | | | | 95°C | | 1min | |  |  |  |  |
| **Cycling x 38** | | | **Denaturation** | | | | | | | 95°C | | 10 secs | |  |  |  |  |
|  |  |  | **Anneal** | | | | | | | 60°C | | 15 secs | |  |  |  |  |
|  |  |  | **Extend** | | | | | | | 72°C | | 10 secs | | Acquire data |  |  |  |
| **Melt Step** | | | **HRM (0.1°C increment, 2s hold)** | | | | | | | 74°C -86°C | | | | Acquire data |  |  |  |
